# Duplication and Reduplication of Papers

**Published:** 1896-04

**Authors:** 


### Editorial.

### DUPLICATION AND REDUPLICATION OF PAPERS.

It is a very natural desire on the part of writers of papers to give them the widest possible circulation, and this desire seems very frequently to be out of all proportion to their value in a scientific sense. It is not unusual to find the same paper published simultaneously in several journals, much to the chagrin of the several editors, who, of course, have not been informed, at the time of the acceptance of the manuscripts, that the writer has forwarded his article all over the United States.

This duplication of papers has become an imposition upon the readers of journals and upon the editorial fraternity, of a very serious character, and it would be well, perhaps, if our contemporaries would keep a list, black list it might be called, of those writers guilty of this, and either bar them entirely from their pages or insist upon an affidavit accompanying each paper that the essay has not been sent to any other journal.

This to the average reader may seem of trifling import, and it is to those journals that, in any event, copy most of their material, but to those mainly devoted to original matter, and which carry a standing announcement that "no papers will be received that have appeared in any other journal published in the country," as the International Dental Journal, it becomes of grave importance affecting their reputation.

It is difficult to understand the real motive for this course in the minds of some very good and original workers, for the results of their labors are so truly of value that were these published even in an obscure journal they would be certain to be copied both at home and abroad; yet while this remains true, we very recently have had evidence of this mistake being made to the injury of the writer and to the discredit of dental journalism. Some of the editors of Western dental periodicals made themselves merry over this, and conspicuously posted the standing announcement in regard to original matter. This is not very hurtful, but it serves to emphasize the fact that it has grown to be a common occurrence, and the victimized are regarded as being fit subjects for the funny men who manufacture paragraphs for that kind of dental literature.

There is another method of publishing, which, while different in motive, is very similar in character, that of having inserted in two journals the proceedings of one society. It is not probable that this will be extended, yet it occurs as the settled policy in one organization, at least, and possibly in others. The actuating motive here is not to give an increased circulation to the report, but to avoid discrimination against either one of two journals in which many of the members have an equal personal interest. This statement is made that it may be understood why a certain prominent dental society has its proceedings published in two journals simultaneously. This, while objectionable, is not a serious matter, and it is also a well-understood business arrangement.

We recently, very reluctantly, accepted an article emanating from a member of one of the State boards. It was published in this journal as the recognized duty of a professional periodical to hear all sides of a question, and it is trusted this literal interpretation will never be departed from, that all sides of a controverted question will be given a hearing and that without regard to the opinions held by the management. This paper it would seem was sent broadcast, and from editorial notices in several journals, it is inferred that the paper was not regarded as worthy of acceptance, and through this, the editor of this journal was saved the annoyance of having a series of repetitions.

If it be regarded as unprofessional to advertise the merits of a certain practice, it must certainly be equally derogatory to the individual to seek notoriety by the procedure mentioned.

The true scientist in any direction rests satisfied with the simple publication of the facts he has been able to discover and then quietly waits for the verdict, satisfied that if he merit it, it will be forthcoming in a degree favorable in proportion to the service rendered. It is this character which should be found worthy of emulation and not that of the empiric who flaunts from the house-top the extent of his knowledge and the value of his productions.

